# Novel Mutation in *CACNA1A* Associated with Activity-Induced Dystonia, Cervical Dystonia, and Mild Ataxia

**DOI:** 10.1155/2021/7797770

**Published:** 2021-08-02

**Authors:** Benjamin Stampfl, Dominic Fee

**Affiliations:** Department of Neurology, Medical College of Wisconsin, Milwaukee, WI, USA

## Abstract

*CACNA1A* encodes the pore-forming *α*1 subunit of the neuronal voltage-gated Cav2.1 (P/Q-type) channels, which are predominantly localized at the presynaptic terminals of the brain and cerebellar neurons and play an important role in controlling neurotransmitter release. Mutations in *CACNA1A* have been associated with several autosomal dominant neurologic disorders, including familial hemiplegic migraine type 1, episodic ataxia type 2 (EA2), and spinocerebellar ataxia type 6. A 37-year-old woman presented with a history of slowly progressive, activity-induced stiffness, and pain in her right leg since age 15 and cervical dystonia since age 20. She denied any right leg stiffness or pain at rest, but when she began to walk, her right foot turned in and her right leg stiffened up. She also had neck pain, stiffness, and spams. There was no family history of similar symptoms. On physical exam, her strength, tone, and reflexes were normal in all extremities at rest. There was mild head titubation and very mild past pointing on finger-to-nose testing. MRI of the brain and spinal cord was unremarkable. This patient's clinical picture was felt to be most consistent with paroxysmal kinesigenic dyskinesia, as she has attacks of dystonia that are triggered by voluntary movement, last from a few seconds to a minute, and are relieved with rest. She was trialed on carbidopa/levodopa without improvement. A dystonia panel showed two potentially pathologic mutations, one in *CACNA1A* and the other in *PNKP*, along with a variant of unknown significance in *ATP7B*. The mutation in *CACNA1A* is C2324 G < A. It is heterozygous, autosomal dominant, and computer modeling suggests pathogenicity. This mutation has not been reported previously and is likely the cause of her paroxysmal dystonia; dystonia is sometimes seen during episodes of ataxia in EA2, and *CACNA1A* knockout mice exhibit dystonia and cerebellar atrophy. After receiving her genetic diagnosis, the patient was trialed on acetazolamide without improvement in her dystonia symptoms. This is the second case report of a patient with cervical dystonia and cerebellar ataxia associated with a mutation in *CACNA1A*.

## 1. Introduction

*CACNA1A* encodes the pore-forming *α*1 subunit of the neuronal voltage-gated Cav2.1 (P/Q-type) channels, which are predominantly localized at the presynaptic terminals of the brain and cerebellar neurons and play an important role in controlling neurotransmitter release [1]. Additionally, it has been shown that *CACNA1A* contains an internal ribosome entry site that initiates the translation of a second protein called *α*1ACT, which is a transcription factor that is important in cerebellar development [2].

Mutations in *CACNA1A* have been associated with several autosomal dominant neurologic disorders, including familial hemiplegic migraine type 1 (FHM1), episodic ataxia type 2 (EA2), and spinocerebellar ataxia type 6 (SCA6) [3]. Familial hemiplegic migraine is characterized by severe headache preceded by an aura with unilateral weakness; the weakness is sometimes accompanied by other neurologic symptoms such as numbness, tingling, hemianopia, ataxia, and aphasia [4]. Patients usually recover fully between episodes, but some develop permanent ataxia. More than 25 pathologic variants in *CACNA1A* have been associated with FHM1, the majority of which are missense, gain-of-function mutations that result in increased calcium influx, and excessive neurotransmission [1]. EA2 is characterized by spells of ataxia lasting hours to days that can be accompanied by vertigo, diplopia, dysarthria, dystonia, and generalized weakness. Between spells, patients often demonstrate persistent nystagmus. In some cases, secondarily progressive ataxia can develop [5]. Unlike FHM1, EA2 is typically caused by loss-of-function mutations that lead to decreased calcium influx. More than 80 pathologic variants in *CACNA1A* have been associated with EA2 [1]. SCA6 is characterized by progressively worsening gait ataxia, dysarthria, dysphasia, diplopia, and mild cognitive impairment. It is caused by 20–33 CAG (polyglutamine) repeats near the C-terminus of the *CACNA1A* gene, which is important for channel function. These repeats may lead to abnormal aggregations of proteins and impaired channel function, contributing to cell dysfunction and death. While SCA6 was previously thought to cause only cerebellar damage, it is now known to cause degeneration of the cortex, thalamus, midbrain, pons, and medulla as well [6]. Dystonia occurs in up to 25% of patients with SCA6. Acetazolamide has been shown to reduce the number of ataxia episodes [7]. There is clinical overlap between FHM1, EA2, and SCA6; about 50% of patients with EA2 also have migraines, and episodic headaches and nausea are also common in SCA6 [1].

## 2. Case

We report the case of a 37-year-old woman with a history of slowly progressive, activity-induced stiffness and pain in her right leg since age 15. Her birth and early childhood history were unremarkable. The patient's symptoms began as a limp in her right leg around age 15; she denied any illnesses, exposures, or trauma around this time. Additionally, at age 20, the patient began having neck pain and stiffness, with occasional neck spasms associated with decreased range of motion.

**Figure 1:**
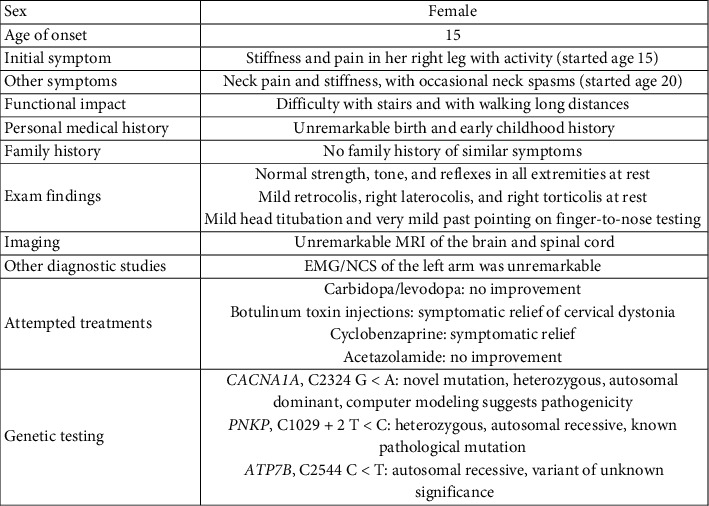
Clinical characteristics and genetic testing.

When evaluated in clinic, she denied any right leg stiffness or pain at rest, but when she began to walk, her right foot turned in and her right leg, knee, proximal thigh, and hip stiffened up. She denied any numbness, tingling, or burning. She denied significant right arm, left arm, or left leg symptoms. The patient denied any gait instability or feeling of imbalance. The patient was using a cane and wearing left and right leg braces, which helped with her walking. She endorsed great difficulty with stairs and with walking long distances. There was no family history of similar symptoms. On physical exam, her strength, tone, and reflexes were normal in all extremities at rest. She also has mild retrocolis, right laterocolis, and right torticolis at rest. There was mild head titubation and very mild past pointing on finger-to-nose testing. There were no other signs of dysmetria or ataxia on exam. MRI of the brain and spinal cord was unremarkable. EMG/NCS of the left arm was unremarkable; lower extremity study was deferred because of recently receiving botulinum toxin injections to the right leg. In light of her history, symptoms, and physical exam, a tentative diagnosis of activity-induced dystonia was made. Given the possibility of it being dopamine-responsive dystonia, the patient was trialed on carbidopa/levodopa without improvement. She also was receiving regular botulinum toxin injections for her cervical dystonia and right leg stiffness, with some improvement in her symptoms. The patient was prescribed cyclobenzaprine as well, with some improvement in her right foot pain and neck spasms. For a table summarizing the patient's clinical characteristics and genetic testing results, please see Figure 1.

A dystonia panel was sent, and it showed two potentially pathologic mutations, one in *CACNA1A* and the other in *PNKP*, along with a variant of unknown significance in *ATP7B*. The mutation in *CACNA1A* is C2324 G < A, protein W775X. It is heterozygous, autosomal dominant, and computer modeling suggests pathogenicity. This mutation has not been reported previously. She also has a heterozygous pathological mutation in *PNKP*, C1029 + 2 T < C, though this is autosomal recessive. The variant of unknown significance in *ATP7B* is C2544, C < T, protein G848G, and is also autosomal recessive. After receiving her genetic diagnosis, the patient was trialed on acetazolamide without improvement in her dystonia symptoms.

## 3. Discussion

This patient's clinical picture is most consistent with paroxysmal kinesigenic dyskinesia, as she has attacks of dystonia that are triggered by voluntary movement, last from a few seconds to a minute, and are relieved with rest [8]. Her paroxysmal dystonia is likely due to her mutation in *CACNA1A*, as it is sometimes seen during episodes of ataxia in EA2 [9], there are other case reports of *CACNA1A* mutations being associated with dystonia, and *CACNA1A* knockout mice (Ca_V_2.1^−/−^) exhibit dystonia and cerebellar atrophy [3]. Dystonia, usually cervical, can occur in EA2 during paroxysmal episodes of ataxia and can become chronic if secondarily progressive ataxia develops. There have also been case reports of patients with EA2 who went on to develop interictal dystonia later in their disease course [9].

Interestingly, there is a 2020 case report of a 62-year-old man with long-standing cerebellar ataxia and cervical dystonia who was found to have a novel mutation in *CACNA1A*. He had a 40-year history of slowly progressive gait instability and a 15-year history of cervical dystonia; he had begun requiring unilateral support 5 years prior to presentation. On exam, he had clear rightward deviation of the head, shoulder flexion, and dysmetria of both the lower limbs and right arm. The patient had an ataxic gait with increased base of support, inability to walk in tandem, and a mild positive Romberg sign. Brain MRI was significant for cerebellar atrophy predominantly affecting the vermis. The novel mutation was a one-nucleotide insertion (c.4056_4057insG), which causes a reading frame shift resulting in a premature stop codon three amino acids after the insertion. This was predicted to cause a truncated and nonfunctional protein [10]. Our patient also had cerebellar ataxia and cervical dystonia, in addition to the paroxysmal dystonia in her right leg. Unlike the other patient, her paroxysmal dystonia appeared first, followed by cervical dystonia, followed by her cerebellar ataxia, which is very mild and associated with subtle findings on neurological exam. However, it is possible that her ataxia could slowly progress over time, as it did in the other patient. Additionally, although her most recent brain MRI at age 37 was normal, she could go on to develop cerebellar atrophy like the other patient. The report of another patient with cervical dystonia and a mutation in *CACNA1A* supports the idea that our patient's cervical dystonia is due to her mutation in *CACNA1A*.

Mice designed to have a homozygous null mutation in *CACNA1A* develop severe ataxia, dystonia, and cerebellar degeneration, supporting the notion that mutations in *CACNA1A* can cause dystonia in humans [3]. This is consistent with the discovery that *CACNA1A* encodes *α*1ACT, a transcription factor that is important for cerebellar development; this transcription factor may also be important for the survival of cerebellar neurons [2]. Additionally, three homogeneous, loss-of-function *CACNA1A* mouse models, *tottering*, *rocker*, and *tottering-4j*, exhibit stress-induced attacks of dystonia, which is thought to be due to cerebellar dysfunction. This provides further evidence in support of our patient's novel *CACNA1A* mutation being the cause of her paroxysmal dystonia. An important limitation to *CACNA1A* mouse models is that a single defective *CACNA1A* allele is sufficient to cause disease in humans, while heterozygous mice are minimally symptomatic (though some aged mice have clear motor deficits) [3].

In our patient, it makes sense that her mutation in *CACNA1A* would be associated with ataxia, given the importance of *CACNA1A* channels to normal cerebellar function; it is less intuitive that such a mutation would led to dystonia. Dystonia has classically been associated with the basal ganglia, especially the striatum. However, more recent research has shown that dystonia is better explained as a network disorder involving the basal ganglia, cortex, thalamus, and cerebellum. In particular, some monogenic animal models of dystonia show changes in cerebellar output that may be associated with basal ganglia dysfunction [11]. Thus, our patient's dystonia may be due in part to cerebellar dysfunction related to her *CACNA1A* mutation.

In summary, we report a novel *CACNA1A* variant associated with activity-induced dystonia of the right leg, cervical dystonia, and mild ataxia.

## Data Availability

No data were used to support this study.

## References

[1] Sutherland H. G., Albury C. L., Griffiths L. R. (2019). Advances in genetics of migraine. *The Journal of Headache and Pain*.

[2] Du X., Wang J., Zhu H. (2013). Second cistron in CACNA1A gene encodes a transcription factor mediating cerebellar development and SCA6. *Cell*.

[3] Pietrobon D. (2010). CaV2.1 channelopathies. *Pflügers Archiv - European Journal of Physiology*.

[4] Di Stefano V., Rispoli M. G., Pellegrino N. (2020). Diagnostic and therapeutic aspects of hemiplegic migraine. *Journal of Neurology, Neurosurgery & Psychiatry*.

[5] Guterman E. L., Yurgionas B., Nelson A. B. (2016). Pearls & oy-sters: episodic ataxia type 2. *Neurology*.

[6] Rentiya Z., Hutnik R., Mekkam Y. Q., Bae J. (2020). The pathophysiology and clinical manifestations of spinocerebellar ataxia type 6. *The Cerebellum*.

[7] Casey H. L., Gomez C. M., Adam M. P., Ardinger H. H., Pagon R. A. (2019). *Spinocerebellar Ataxia Type 6*.

[8] Méneret A., Roze E. (2016). Paroxysmal movement disorders: an update. *Revue neurologique*.

[9] Indelicato E., Boesch S. (2021). From genotype to phenotype: expanding the clinical spectrum of CACNA1A variants in the era of next generation sequencing. *Frontiers in Neurology*.

[10] Fuerte-Hortigón A., Pérez-Noguera R., Dotor García-Soto J., Royo Boronat I., Álvarez de Andrés S., García-Moreno J. M. (2020). Novel CACNA1A variant may cause cervical dystonia and cerebellar ataxia syndrome. *Journal of the Neurological Sciences*.

[11] Schirinzi T., Sciamanna G., Mercuri N. B., Pisani A. (2018). Dystonia as a network disorder: a concept in evolution. *Current Opinion in Neurology*.

